# Phototropin 2 mediates daily cold priming to promote light responses in Arabidopsis

**DOI:** 10.1093/jxb/eraf040

**Published:** 2025-01-30

**Authors:** Minoru Noguchi, Issei Keino, Hitomi Takahashi, Shota Yamauchi, Mami Fujisawa, Ken Haga, Tatsuya Sakai, Atsushi Takemiya, Yutaka Kodama

**Affiliations:** Center for Bioscience Research and Education, Utsunomiya University, Tochigi 321-8505, Japan; Graduate School of Regional Development and Creativity, Utsunomiya University, Tochigi 321-8505, Japan; Center for Bioscience Research and Education, Utsunomiya University, Tochigi 321-8505, Japan; Graduate School of Regional Development and Creativity, Utsunomiya University, Tochigi 321-8505, Japan; Center for Bioscience Research and Education, Utsunomiya University, Tochigi 321-8505, Japan; Department of Biology, Graduate School of Sciences and Technology for Innovation, Yamaguchi University, Yamaguchi 753-8512, Japan; Center for Bioscience Research and Education, Utsunomiya University, Tochigi 321-8505, Japan; Department of Applied Chemistry, Faculty of Fundamental Engineering, Nippon Institute of Technology, Saitama 345-8501, Japan; Graduate School of Science and Technology, Niigata University, Niigata 950-2181, Japan; Department of Biology, Graduate School of Sciences and Technology for Innovation, Yamaguchi University, Yamaguchi 753-8512, Japan; Center for Bioscience Research and Education, Utsunomiya University, Tochigi 321-8505, Japan; Graduate School of Regional Development and Creativity, Utsunomiya University, Tochigi 321-8505, Japan; University of Glasgow, UK

**Keywords:** Arabidopsis, blue light, cold, photoreceptor, phototropism, stomatal opening, thermosensor

## Abstract

Organisms adapt to predictable environmental changes via a biological mechanism called priming. Phototropin is a plant-specific blue light photoreceptor that mediates daily light-induced responses, such as chloroplast relocation, stomatal opening, and phototropism, to optimize photosynthesis. Phototropin also functions as a thermosensor for chloroplast relocation that may sense daily temperature decreases at night, thereby modulating light-induced responses at dawn; however, this hypothesis has not yet been fully explored. Here, we revealed that phototropin mediates daily cold priming to promote stomatal opening and phototropism in Arabidopsis under dawn-mimicking conditions. A cold pretreatment in the dark enhanced subsequent blue light-induced stomatal opening and phototropism at normal temperatures, suggesting that daily cold priming is involved in these physiological responses. Arabidopsis has two phototropin proteins (phot1 and phot2), and we showed that phot2 clearly mediates cold priming of stomatal opening and phototropism. Cold priming appears to be based on phototropin-mediated thermosensing just before dawn, which plants use to optimize their light-induced responses in anticipation of dawn.

## Teaser

Phototropin 2 functions as both photoreceptor and temperature sensor in Arabidopsis, priming blue light-mediated stomatal opening and phototropism at dawn based on pre-dawn temperatures.

## Introduction

Priming is a biological phenomenon by which organisms adjust their physiological responses in anticipation of future environmental conditions. For example, anticipating light conditions at dawn would allow plants to adjust their metabolism for optimal photosynthesis in the morning. Temperature could provide a key input in priming, as temperatures rise during the first half of the day and fall during the evening and at night; moreover, cloudy nights are warmer than clear nights because clouds block radiative cooling. However, whether plants sense daily night-time cold conditions to prime their physiological responses at dawn remains unclear.

Phototropins (phots) are plant-specific blue light (BL) photoreceptors with two photoreceptive light-oxygen-voltage (LOV1 and LOV2) domains at their N terminus and a kinase domain at the C terminus (Christie, 2007). Each LOV domain undergoes a photocycle reaction as part of BL perception; under dark conditions, each LOV domain associates with the chromophore flavin mononucleotide (FMN) (inactive form of the LOV domain); upon excitation with BL, the LOV domain forms a covalent bond with the FMN (active form of the LOV domain). The activated form stimulates the kinase domain, leading to phot autophosphorylation. In the photocycle reaction, the active form of each LOV domain reverts to the inactive form; this reversion is called thermal reversion to indicate its temperature-regulated nature. The protein sequence and photocycle properties of phot are widely conserved in land plants and green algae. One process phot contributes to is photosynthetic optimization; for example, phot mediates BL-induced chloroplast relocation dependent on BL intensity (Christie, 2007). In the case of the liverwort *Marchantia polymorpha*, which has a single-copy gene for phot, when a cell is exposed to weak BL, phot is photoactivated through either the LOV1 or the LOV2 domain, and chloroplasts move toward the weak BL signal to maximize their light absorption (accumulation response) (Sakai *et al.*, 2001; Gotoh *et al.*, 2018; Kato *et al.*, 2021). When phot is excited by strong BL, the proportion of phot photoactivated through LOV2 increases, and chloroplasts move away from the strong BL signal to mitigate photodamage (avoidance response) (Jarillo *et al.*, 2001; Kagawa *et al.*, 2001; Kasahara *et al.*, 2002; Kato *et al.*, 2021). Phot is also reported to act as a thermosensor in the modulation of this chloroplast relocation response (Kodama *et al.*, 2008; Fujii *et al.*, 2017). When the cell receives weak BL under cold conditions, the proportion of phot photoactivated through LOV2 increases due to the slower thermal reversion rate of LOV2, resulting in a light avoidance response even under weak BL conditions (Fujii *et al.*, 2017). This phot-mediated cold-induced light avoidance response occurs in various plant species, including *M. polymorpha* and the angiosperm Arabidopsis (*Arabidopsis thaliana*), suggesting that the thermosensing function of phot is likely evolutionarily conserved (Kodama *et al.*, 2008; Ogasawara *et al.*, 2013; Fujii *et al.*, 2017).

Arabidopsis has two phots (phot1 and phot2), with phot2 being essential for the temperature dependence of the chloroplast relocation response, suggesting that it may act as the main thermosensor (Fujii *et al.*, 2017). In addition, phot1 and phot2 mediate not only chloroplast relocation, but also stomatal opening and phototropism (Kinoshita *et al.*, 2001; Sakai *et al.*, 2001). Stomatal opening increases the uptake of carbon dioxide, while phototropism increases light absorption by the photosynthetic apparatus. Phot1 and phot2 redundantly mediate these responses and activate downstream factors such as BLUE LIGHT SIGNALING 1 (BLUS1) for stomatal opening, ROOT PHOTOTROPISM 2 (RPT2) and NONPHOTOTROPIC HYPOCOTYL 3 (NPH3) for phototropism (Motchoulski and Liscum, 1999; Sakai *et al.*, 2000; Takemiya *et al.*, 2013a). As temperatures decrease, however, the photoactivation of phot is promoted (because thermal reversion is inhibited), while almost all other cellular biochemical activities slow down. In such a case, the reduced cellular activity would counteract the effect of cold-induced phot activation, obscuring any potential involvement of phot1 and phot2 as thermosensors in stomatal opening and phototropism.

We previously proposed that the BL-receptive thermosensor phot functions at dawn because early morning light is rich in blue (short) wavelengths (Fujii *et al.*, 2017; Noguchi and Kodama, 2022). Based on this hypothesis, in this study, we developed a culture system mimicking dawn to explore stomatal opening and phototropism in wild type (WT) and *phot* mutants of Arabidopsis. We determined that cold-mediated priming (cold priming) of BL-induced stomatal opening and phototropic responses occurs at dawn and requires phot2.

## Materials and methods

### Plant material and growth conditions

To study stomatal opening and phototropism, the Arabidopsis wild type (WT, Col-0 *gl-1*) and *phot1-5*, *phot2-1*, and *phot1-5 phot2-1* mutants were used.

To analyse stomatal opening, seeds were surface sterilized, sown on half-strength Murashige and Skoog (MS) medium [solidified with 0.5% (w/v) gellan gum], stratified in the dark at 4 °C for 3 d, and cultured for 12 d under 50 µmol m^–2^ s^–1^ continuous white light (FL15EX-N-Z; Toshiba Lighting & Technology Corporation, Tokyo, Japan) at 22 °C in an incubator (LH-240SP; Nippon Medical & Chemical Instruments Co., Ltd, Osaka, Japan). The light intensity was measured with a light meter (LI-250A; LI-COR Biosciences, Lincoln, NE, USA). The cultured seedlings were transplanted to a mixture of vermiculite–garden soil (2:1; v/v) and cultivated for 3–5 weeks under a 16 h light/8 h dark cycle at 22 °C in an incubator (LH410SP; Nihon Medical & Chemical Instruments Co.).

To analyse phototropism, seeds were surface sterilized, sown on half-strength MS medium [solidified with 0.5% (w/v) gellan gum], stratified in the dark at 4 °C for 3 d, and germinated under 50 µmol m^–2^ s^–1^ of continuous white light at 22 °C for 24 h in an incubator (LH-240SP; Nippon Medical & Chemical Instruments Co.). The germinated seeds were vertically cultured in an incubator (IJ102; Yamato Scientific Co., Ltd, Japan) in the dark at 22 °C for 3 d to obtain etiolated seedlings (~5–8 mm long) (Zhu *et al.*, 2021).

### Stomatal opening

To measure stomatal opening, each leaf was cut into pieces (~5×5 mm), and the tissue was degassed in reaction buffer (50 mM KCl, 0.1 mM CaCl_2_, and 25 µM Bis–Tris, pH 6.5). The samples were pre-incubated in reaction buffer in the dark at 7 °C for 90 min in an incubator (IJ101; Yamato Scientific Co.). To mimic night-time, the pre-incubated tissue was further incubated in the dark at 22 °C or 7 °C for 1 h. The abaxial side of the leaf piece was irradiated with 70 µmol m^–2^ s^–1^ BL or 50 µmol m^–2^ s^–1^ red light (RL) (ISL-150X150-RHB; CCS Inc., Kyoto, Japan) at 22 °C. The light intensity was determined based on the CO_2_ assimilation rate measured using a portable photosynthesis system (LI-6800; LI-COR Biosciences). To avoid photoperiodic effects, each treatment was conducted at the same hour [pre-incubation in the dark at 7 °C for 90 min (from 09.30 to 11.00 h), incubation in the dark at 22 °C or 7 °C for 1 h (from 11.00 to 12.00 h), and light irradiation at 22 °C (from 12.00 h)]. The stomata were observed under a light microscope (DM5000B; Leica Microsystems, Wetzlar, Germany), with images captured at 10 min intervals using a digital camera (DP72; Olympus, Tokyo, Japan). Using these images, the apertures of 10 stomata were measured using ImageJ software (https://imagej.net/ij/) (Schneider *et al.*, 2012), and the average value was calculated. This experiment was performed five times, and the five average values were used for statistical analysis.

### Phototropism

To mimic night-time, dark-grown (etiolated) seedlings were incubated in the dark at 22 °C or 7 °C for 4 h in an incubator (IJ102; Yamato Scientific Co.). An additional 1 h dark incubation at 22 °C after the 4 h dark incubation at 7 °C was also performed in this incubator. The incubated seedlings were irradiated with 10 µmol m^–2^ s^–1^ of BL (ISL-150X150-RHB; CCS Inc.) in an incubator (LH-410SP; Nippon Medical & Chemical Instruments Co.). The BL was provided from the side. A camera (GoPro HERO9 Black; GoPro Inc., San Mateo, CA, USA) was used to take images at 5 min intervals. Using these images, the curvatures of 7–14 hypocotyls were measured using ImageJ, and the average value was calculated. This experiment was performed five times, and the five average values were used for statistical analysis.

### Immunoblotting

Guard cell protoplasts were prepared from leaves and subjected to immunoblotting analyses of BLUS1 and phospho-BLUS1 as previously described (Ueno *et al.*, 2005, Takemiya, 2013a, b). BLUS1 and BLUS1 phosphorylated at Ser-348 were detected using anti-BLUS1 and anti-phospho-BLUS1 polyclonal antibodies from rabbit, respectively, as previously described (Takemiya *et al.*, 2013a).

RPT2 and NPH3 were detected by immunoblotting using anti-RPT2 and anti-NPH3 rabbit polyclonal antibodies as the primary antibodies at dilutions of 1:1000 and 1:500, respectively. The anti-RPT2 and anti-NPH3 antibodies were previously described (Inada *et al.*, 2004; Tsuchida-Mayama *et al.*, 2008). The membranes were incubated with the above-mentioned goat anti-rabbit IgG (H+L) secondary antibody at a dilution of 1:1000 (for RPT2) or 1:500 (for NPH3) with Can Get Signal (TOYOBO, Osaka, Japan). Electrophoretic mobility shift assays for NPH3 were performed using band shift gels. The proteins were resolved in 8% (w/v) polyacrylamide gels (acrylamide:*N*,*N*′-methylenebisacrylamide =29.9:1). Chemiluminescence signals were analysed using ECL-Select Western Blotting Detection Reagent (Cytiva, Marlborough, MA, USA) and a LuminoGraph III Chemiluminescent Imaging System (ATTO, Tokyo, Japan).

### Analysis of the photocycles of the LOV domains of phot1 and phot2

DNA fragments encoding the LOV2 domains of phot1 and phot2 (LOV2^phot1^ and LOV2^phot2^, respectively) were amplified by PCR using an Arabidopsis cDNA library as a template. Primers for LOV2^phot1^ and for LOV2^phot2^ are given in Supplementary Table S1. The resulting DNA fragments were cloned into pDONR207 by BP reaction (Gateway cloning technology; Thermo Fisher Scientific, Waltham, USA), yielding pDONR207-LOV2^phot1^ and pDONR207-LOV2^phot2^. These constructs were mixed with the destination vector pKM596 (Addgene: 8837), and an LR reaction (Gateway cloning technology) was performed to generate pKM596-LOV2^phot1^ and pKM596-LOV2^phot2^. The pKM596-LOV2^phot1^ and pKM596-LOV2^phot2^ plasmids were transformed into *Escherichia coli* strain Rosetta-gami 2 (Novagen, San Diego, CA, USA). Maltose-binding protein (MBP)-tagged recombinant LOV2^phot1^ and LOV2^phot2^ (MBP-LOV2^phot1^ and MBP-LOV2^phot2^, respectively) were produced in the transformed *E. coli* and purified as reported previously (Fujii *et al.*, 2017).

The amounts of the active forms of LOV2^phot1^ and LOV2^phot2^ under BL conditions were determined by monitoring fluorescence at 500 nm originating from the inactive form (Kato *et al.*, 2021). The fluorescence intensity at 500 nm was measured at 22 °C or 7 °C under excitation at 450 nm using a fluorescence spectrophotometer (F2700; Hitachi High-Tech Co., Tokyo, Japan). The sample temperature was controlled as reported previously (Kato *et al.*, 2021). Before measuring fluorescence, recombinant purified MBP-LOV2^phot1^ and MBP-LOV2^phot2^ were incubated at 22 °C in the dark for 30 min to convert the LOV domains to the inactive form and further incubated at 22 °C or 7 °C for 15 min. When MBP-LOV2^phot1^ and MBP-LOV2^phot2^ were irradiated with 12 or 120 µmol m^–2^ s^–1^ excitation light at 450 nm, the fluorescence initially exhibited at maximum intensity (*F*_a_, fluorescence produced when all LOV domains are in the inactive form) and gradually decreased. After approximately 10 min, the fluorescence intensity became constant, indicating that the photocycle had reached a state of photoequilibrium (*F*_e_). The proportion of LOV domains in the active form was calculated as [(*F*_a_−*F*_e_)/*F*_a_]×100.

The kinetics of the thermal reversion of the active form to the inactive form of LOV2^phot1^ and LOV2^phot2^ in the dark were analysed as described previously (Kato *et al.*, 2021).

## Results

### Phot2 mediates cold priming of stomatal opening

**Fig. 1.**
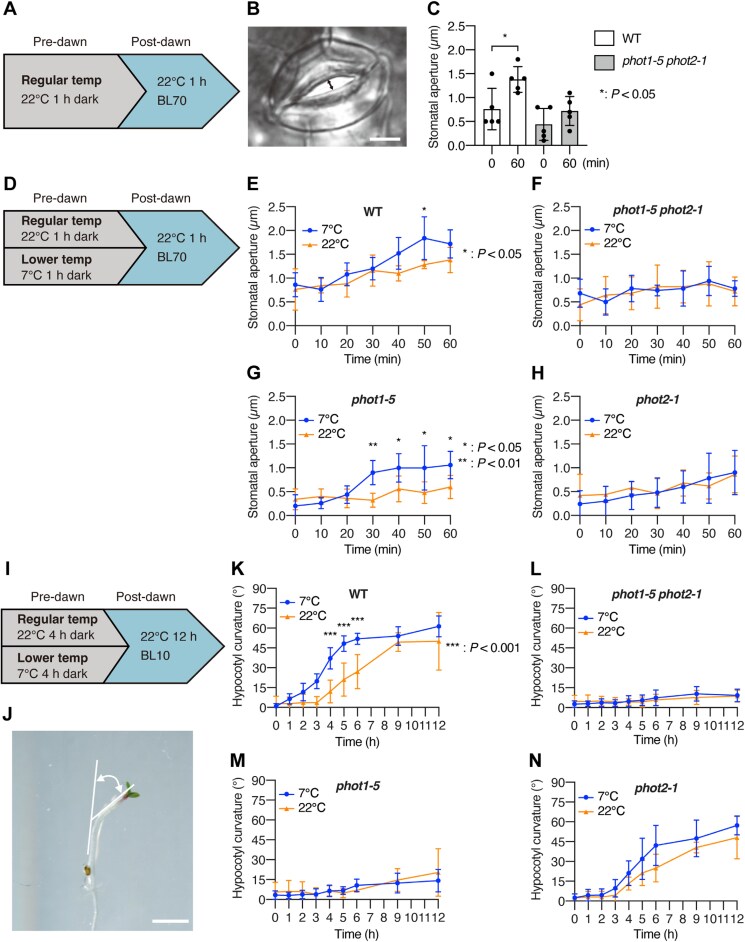
Phot2 mediates the cold priming of stomatal opening and phototropism in Arabidopsis. (A) Diagram of dawn-mimicking conditions used to induce stomatal opening using a 22 °C pre-dawn treatment. Temp, temperature; BL70, blue light at 70 µmol m^–2^ s^–1^. (B) Representative image used to measure stomatal aperture. The double-headed arrow indicates the stomatal aperture. Scale bar, 5 µm. (C) Stomatal opening in the wild type (WT) and the *phot1-5 phot2-1* double mutant after dawn under the dawn-mimicking conditions illustrated in (A) (*n*=5). Asterisks indicate significant differences (Student’s *t*-test). (D) Diagram of the dawn-mimicking conditions used to induce stomatal opening with a 7 °C (cold priming) or 22 °C (control) pre-dawn treatment. (E–H) Stomatal opening with cold priming in the WT (E), *phot1-5 phot2-1* (F), *phot1-5* (G), and *phot2-1* (H) under the dawn-mimicking conditions illustrated in (D) (*n*=5). (I) Diagram of dawn-mimicking conditions used to induce phototropism with 10 µmol m^–2^ s^–1^ blue light (BL10). (J) Representative image of the measurement of hypocotyl curvature. The double-headed curved arrow indicates the angle of curvature. Scale bar, 5 mm. (K–N) Phototropism and cold priming in the WT (K), *phot1-5 phot2-1* (L), *phot1-5* (M), and *phot2-1* (N) after dawn under the dawn-mimicking conditions illustrated in (I) (*n*=5). Values are means ±SD (C, E–H, K–N). Asterisks indicate significant differences (Šidák’s multiple comparisons test) (E–H, K–N).

To examine stomatal opening under dawn-mimicking conditions, we pretreated Arabidopsis WT plants in the dark for 1 h to mimic the time before dawn, followed by treatment under BL (70 µmol m^–2^ s^–1^) for 1 h to mimic the time after dawn, all at 22 °C (Fig. 1A). After dawn, stomatal aperture (Fig. 1B) was greater than in the preceding period of darkness (Fig. 1C). Because stomatal opening is redundantly regulated by phot1 and phot2 under standard experimental conditions (Kinoshita *et al.*, 2001), we used a *phot1 phot2* double mutant (*phot1-5 phot2-1*) as a control. The double mutant did not undergo stomatal opening after dawn (Fig. 1C).

A previous study suggested that phot2 functions as a thermosensor to mediate chloroplast relocation (Fujii *et al.*, 2017). We therefore reasoned that phot1 and/or phot2 might be involved in the pre-dawn cold priming of post-dawn stomatal opening. To test this hypothesis, we analysed stomatal opening under dawn-mimicking conditions following cold priming. We pretreated plants with control (22 °C) or a lower temperature (7 °C) before dawn to examine the cold priming of stomatal opening (Fig. 1D). In the WT, the degree of stomatal opening was significantly higher at 50 min after dawn following pre-dawn treatment at 7 °C versus 22 °C (Fig. 1E), indicating that cold priming of stomatal opening took place during the hour-long timeframe of the cold pretreatment. The *phot1-5 phot2-1* mutant did not exhibit cold priming of stomatal opening (Fig. 1F). To determine whether phot1, phot2, or both mediate this cold priming, we performed the same experiment using *phot1* and *phot2* single mutants (*phot1-5* and *phot2-1*, respectively) (Fig. 1G, H). We observed the cold priming effect in the *phot1-5* mutant, but not in *phot2-1* (Fig. 1G, H). This finding indicates that phot2, but not phot1, mediates the hour-scale cold priming of stomatal opening in Arabidopsis.

Stomatal opening can be induced by photosynthesis in a phot-independent manner (Kinoshita *et al.*, 2001). To test whether photosynthesis mediates the cold priming of stomatal opening, we used red light (RL) as a post-dawn light source, which stimulates photosynthesis but not phot1 or phot2 activity. We set the RL intensity (50 µmol m^–2^ s^–1^) to achieve a level of photosynthesis (as measured by CO_2_ assimilation) equal to that obtained under our BL conditions (70 µmol m^–2^ s^–1^) (Supplementary Fig. S1A, B). Under dawn-mimicking conditions with RL (Supplementary Fig. S1C), the degree of stomatal opening in WT plants subjected to 7 °C pre-dawn treatment was comparable to that of plants subjected to 22 °C pre-dawn treatment (Supplementary Fig. S1D). We conclude that the cold priming of stomatal opening is not mediated via photosynthesis.

In summary, light-induced stomatal opening is mediated by phot1, phot2, and photosynthesis, but cold priming is mediated only by phot2.

### Phot2 mediates cold priming of phototropism

We analysed phototropism under dawn-mimicking conditions by pretreating etiolated Arabidopsis seedlings in the dark at 22 °C or 7 °C for 4 h to mimic pre-dawn, followed by 22 °C treatment under BL (10 µmol m^–2^ s^–1^) for 12 h to mimic the time after dawn (Fig. 1I). To analyse phototropism, we measured the hypocotyl curvature of these seedlings as described previously (Zhu *et al.*, 2021) (Fig. 1J). Under dawn-mimicking conditions, both WT and *phot2-1* exhibited phototropism, while *phot1-5* and *phot1-5 phot2-1* did not (Fig. 1K–N). Although the degree of phototropism was slightly higher in the *phot1-5* single mutant than in the *phot1-5 phot2-1* double mutant (Fig. 1L, M), both *phot1-5* and *phot1-5 phot2-1* showed severe defects in phototropism. Therefore, under dawn-mimicking conditions, phototropism is mainly mediated by phot1, but not phot2.

The degree of phototropism was significantly higher in WT seedlings exposed to 7 °C pre-dawn pretreatment than to 22 °C pretreatment until 6 h after dawn (Fig. 1K). By contrast, *phot2-1* did not exhibit a statistically significant promotion of phototropism under 7 °C versus 22 °C pretreatment (Fig. 1N), although it showed a similar trend to WT (Fig. 1K). We detected no phototropism in *phot1-5* or *phot1-5 phot2-1*, even after 7 °C pretreatment (Fig. 1L, M). These findings demonstrate that the light sensing that induces phototropism in Arabidopsis seedlings is mainly mediated by phot1 and that phot2 mediates the cold priming of this response.

### The activation of phot2 occurs in a temperature-dependent manner

The above results suggest that phot2, not phot1, is activated for cold priming under dawn-mimicking conditions. Just after dawn, because there is a slight lag between changes in ambient temperatures and those inside the plant, phot1 and phot2 are likely still at the preincubation temperature (22 °C or 7 °C) when they first perceive BL.

**Fig. 2.**
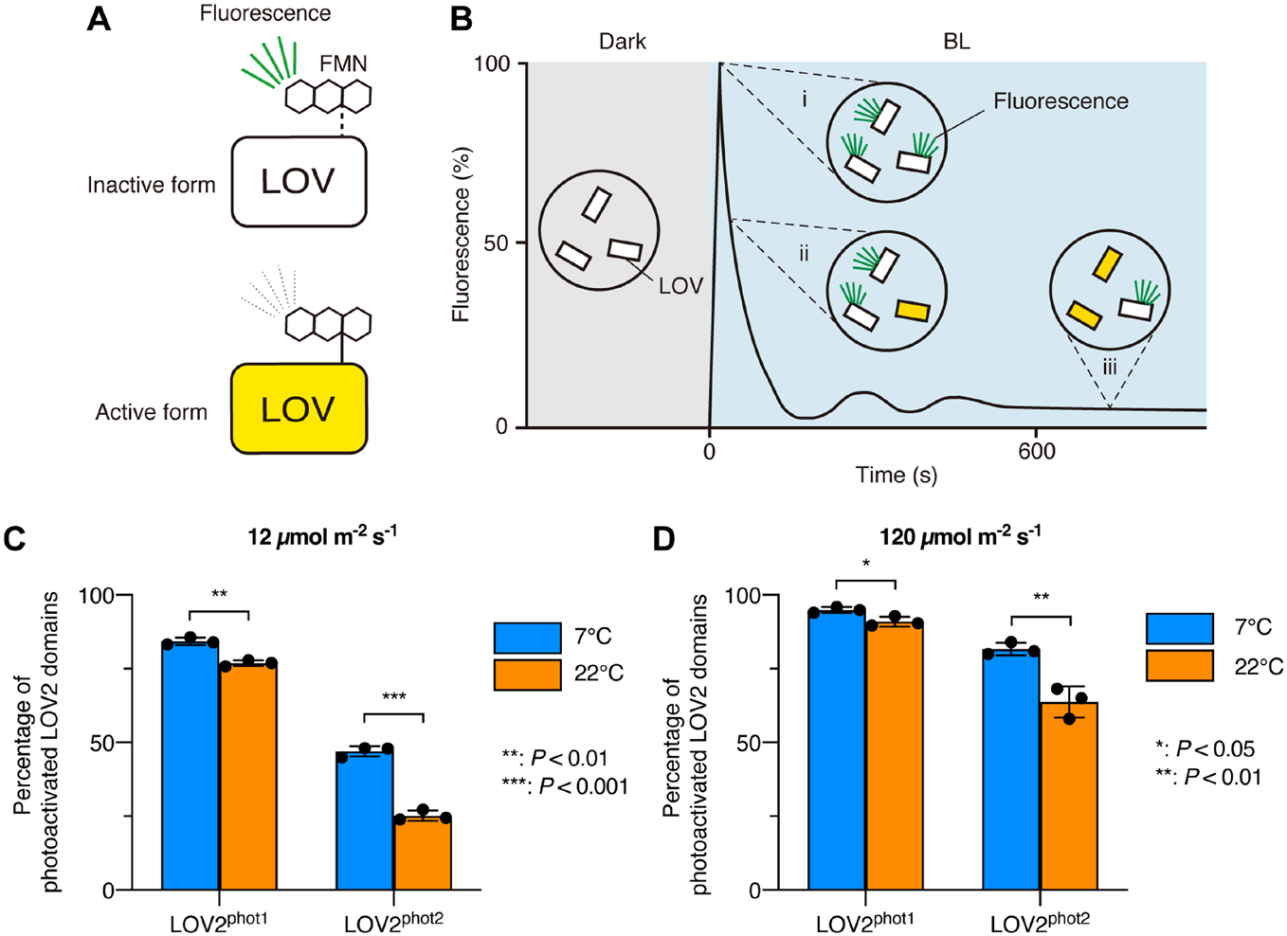
The levels of photoactive phot2 greatly increase under low-temperature conditions. (A) Diagram of the fluorescence from the inactive form and the non-fluorescent active form of the LOV domains. The inactive form emits fluorescence (peaking at 500 nm) with an excitation at 450 nm. (B) Diagram of the change in fluorescence intensity originating from the inactive form of the LOV domain. (i) Fluorescence from all LOV domains. (ii) Decline of fluorescence. (iii) Equilibrium state of fluorescence. BL, blue light. (C) Percentage of the phot1 and phot2 LOV2 domains in the active form at photoequilibrium under weak BL conditions (12 µmol m^–2^ s^–1^) at 7 °C or 22 °C (*n*=3). (D) Percentage of the phot1 and phot2 LOV2 domains in the active form at photoequilibrium under strong BL conditions (120 µmol m^–2^ s^–1^) at 7 °C or 22 °C (*n*=3). Values are means ±SD (C, D). Asterisks indicate significant differences (Student’s *t*-test) (C, D).

Phot proteins contain two photoreceptive domains, LOV1 and LOV2, at their N termini (Christie, 2007), and the activation level of phot depends on the level of its active LOV2 domains (Christie, 2007; Fujii *et al.*, 2017). Accordingly, we developed an *in vitro* fluorescence-based assay to measure the amounts of active LOV2 domains from phot1 and phot2 (LOV2^phot1^ and LOV2^phot2^) under dawn-mimicking conditions. When LOV domains are excited at 450 nm, the inactive form emits fluorescence that originates from the FMN chromophore, but the active form does not (Kato *et al.*, 2021) (Fig. 2A). By measuring the fluorescence intensity, we determined the amount of the active form present under each condition using recombinant purified proteins in solution (Fig. 2B). Briefly, in the dark (i.e. before dawn), all LOV domains revert to their inactive form (Fig. 2B). Upon exposure to BL (i.e. at dawn), the initial fluorescence intensity (*F*_a_) from the inactive forms (fluorescent) can be regarded as the apparent total amount of LOV domain present in the solution (Fig. 2B, i). Over time, the inactive form (fluorescent) gradually converts to the active form (non-fluorescent), leading to a decline in fluorescence intensity (Fig. 2B, ii). A fraction of the active form (non-fluorescent) thermally reverts to the inactive form (fluorescent), reaching an equilibrium state (*F*_e_) (Fig. 2B, iii). The proportion of the active form at the equilibrium state can be estimated using the formula [(*F*_a_−*F*_e_)/*F*_a_]×100.

We used recombinant LOV2^phot1^ and LOV2^phot2^ proteins produced in *E. coli*, monitored their fluorescence intensity, and calculated the amounts of the active forms of LOV2^phot1^ and LOV2^phot2^ under two different BL intensities (12 or 120 µmol m^–2^ s^–1^) at 22 °C and 7 °C (Fig. 2C, D). Under both BL intensities, the amounts of the active forms of LOV2^phot1^ and LOV2^phot2^ were significantly higher at 7 °C than at 22 °C (Fig. 2C, D). This *in vitro* assay suggested that the amounts of the active forms of LOV2^phot1^ and LOV2^phot2^ tend to change markedly in a temperature-dependent manner under the two BL intensities. Importantly, the difference in activation level between 7 °C and 22 °C was greater for phot2 than for phot1 (Fig. 2C, D).

Taken together, these results suggest that under dawn-mimicking conditions, low temperature exposure at pre-dawn and BL irradiation at dawn greatly increase the amounts of active LOV2 domains within phot2, resulting in the promotion of stomatal opening and phototropism in Arabidopsis.

### Signaling pathways mediated by phot2, not phot1, are activated for cold priming

**Fig. 3.**
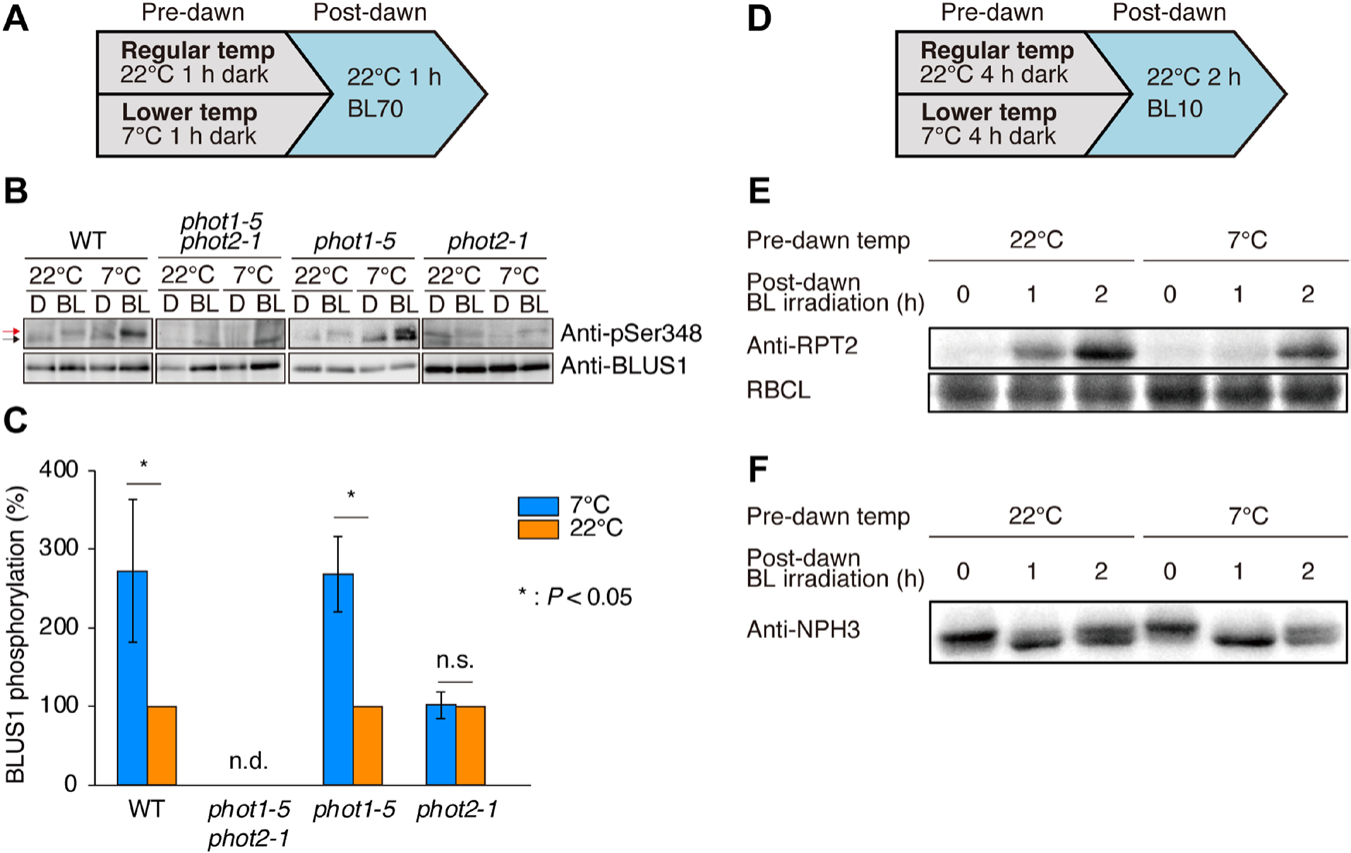
Cold priming is mediated by a phot2-dependent and phot1-independent signaling pathway. (A–C) Phosphorylation levels of BLUS1 in guard cells under dawn-mimicking conditions. (A) Diagram of the experimental design used to analyse BLUS1 proteins. Immunoblotting (B) and quantification of signal intensity (C) (*n*=3, biological replicates) are shown. Red and black arrows (B) indicate phosphorylated BLUS1 signals and non-specific signals, respectively. Asterisks in (C) indicate significant differences (Student’s *t*-test); n.d., not detected; n.s., no significant difference. Values are means ±SD (C). (D–F) Accumulation pattern of RPT2 and dephosphorylation of NPH3 in etiolated seedlings under dawn-mimicking conditions. (D) Diagram of the experimental design used to analyse RPT2 and NPH3 abundance. (E, F) Immunoblotting analyses of RPT2 (E) and NPH3 (F) performed with anti-RPT2 and anti-NPH3 antibodies, respectively. Coomassie brilliant blue staining of ribulose-1,5-bisphosphate carboxylase/oxygenase large subunit (RBCL) is shown as the loading control (E). BL, blue light; temp, temperature.

Next, we analysed proteins known to regulate stomatal opening and phototropism. The guard cell kinase BLUS1 acts downstream of phot1 and phot2 in stomatal opening (Takemiya *et al.*, 2013a). Phot contains a kinase domain at the C terminus by which BLUS1 is phosphorylated following the photoactivation of phot (Christie, 2007; Takemiya *et al.*, 2013a). We used immunoblotting analysis to measure BLUS1 phosphorylation levels in guard cell protoplasts and observed that BLUS1 was phosphorylated under dawn-mimicking BL conditions at 22 °C in the WT and the *phot1-5* and *phot2-1* single mutants but not the *phot1-5 phot2-1* double mutant (Fig. 3A–C). Moreover, BLUS1 phosphorylation at 1 h after dawn was enhanced by cold treatment (7 °C) before dawn; this enhancement was abolished in *phot2-1* and *phot1-5 phot2-1* but not in *phot1-5* (Fig. 3B, C). These results indicate that both phot1 and phot2 activate the signaling pathway for stomatal opening via BLUS1 phosphorylation under BL conditions in Arabidopsis guard cells under dawn-mimicking conditions, while only phot2 further activates this pathway when the guard cells are pre-incubated at low temperature.

No phot2-related proteins that regulate phototropism have been reported (Haga and Sakai, 2023). By contrast, the phot1-related signaling pathway for phototropism is known to involve RPT2 and NPH3 (Haga and Sakai, 2023). RPT2 binds to phot1 and suppresses its autophosphorylation to promote phototropism (Kimura *et al.*, 2020), while NPH3 is dephosphorylated dependent on a phot1-mediated phosphorylation (Tsuchida-Mayama *et al.*, 2008; Sullivan *et al.*, 2021). To explore whether and how these proteins act under dawn-mimicking conditions, we analysed RPT2 abundance and NPH3 phosphorylation levels. RPT2 levels decreased slightly at 7 °C compared with 22 °C (Fig. 3D, E), and the phosphorylation pattern of NPH3 was comparable at 7 °C and 22 °C (Fig. 3D, F). In this context, the signaling pathway mediated by RPT2 and NPH3 might not contribute to the cold priming of phototropism at 7 °C. Based on the above results, the cold priming of phototropism appears to be mediated by an unknown signaling pathway via phot2, but not by the phot1-related signaling pathway.

### Thermal memory for cold priming is rewritable

**Fig. 4.**
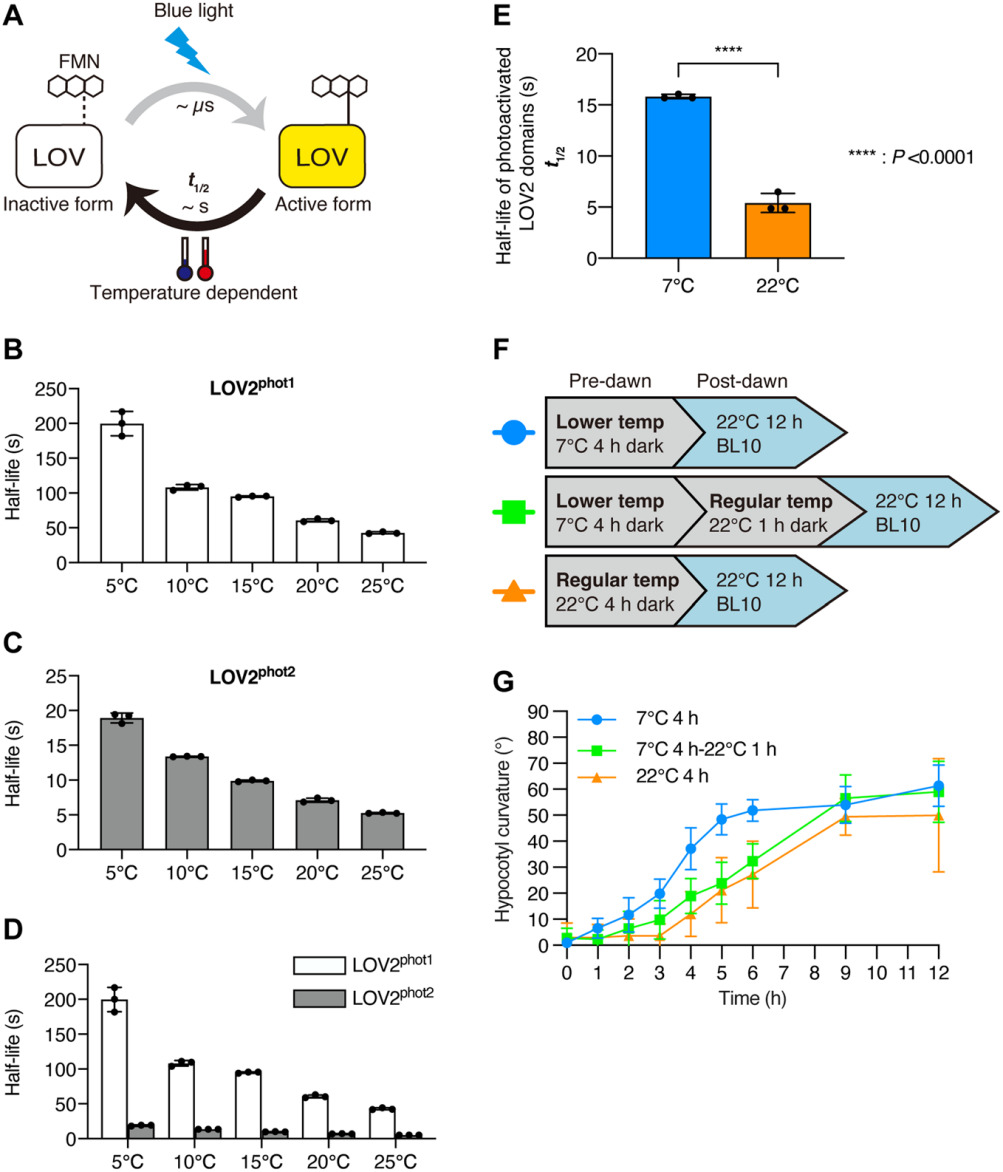
Rewriting of thermal memory before dawn. (A) Diagram of the photocycle of the LOV domain. The active form reverts to its inactive form in a temperature-dependent manner. The reversion rate can be estimated as the half-life (*t*_1/2_) of the active form. (B) Half-lives of the active form of LOV2^phot1^ at various temperatures (*n*=3). (C) Half-lives of the active form of LOV2^phot2^ at various temperatures (*n*=3). (D) Half-lives of the active form of LOV2^phot1^ (B) and LOV2^phot2^ (C), plotted on the same scale. (E) Half-lives of the active form of LOV2^phot2^ at 7 °C and 22 °C (*n*=3). Asterisks indicate significant differences (Student’s *t*-test). (F) Diagram of the dawn-mimicking conditions used to induce phototropism with 10 µmol m^–2^ s^–1^ of blue light (BL10). Before dawn, three different temperature conditions were used: (i) 7 °C for 4 h; (ii) 7 °C for 4 h, followed by 22 °C for 1 h; and (iii) 22 °C for 4 h. Temp, temperature. (G) Phototropism and cold priming in the WT after dawn under the dawn-mimicking conditions illustrated in (F) (*n*=5). Values are means ±SD (B–E, G).

To date, cold priming has been described as a day-scale thermal memory response (e.g. DNA methylation) and an important mechanism for acclimation during seasonal temperature changes (Wanner and Junttila, 1999; Van Der Lans *et al.*, 2013). In the above experiments, we revealed that hour-scale phot2-mediated cold priming promotes light responses in Arabidopsis, pointing to a phot2-based thermal memory involving its thermal reversion (i.e. biochemical reaction). Considering the thermal reversion-based thermosensing ability of LOV2 within phot, we concluded that the thermal reversion of LOV2^phot2^ is the basis for phot2-based thermal memory. To further analyse the temperature dependence of the reversion of LOV2^phot2^, we measured the half-life of the active form of recombinant purified LOV2^phot2^ under controlled temperatures (Fig. 4A). The half-lives of LOV2^phot2^ were shorter than those of LOV2^phot1^ in the temperature range of 5–25 °C (Fig. 4B–D); the half-lives of LOV2^phot2^ at 7 °C and 22 °C were 15.8 and 5.4 s, respectively (Fig. 4E). These results suggest that phot2-based thermal memory operates on a timescale of less than 1 min.

Given this phot2-based short thermal memory, we examined whether phot2-mediated cold priming is based on the temperature immediately preceding dawn. As mentioned above, since *in planta* temperatures do not change immediately after ambient temperatures change, we explored phototropism in plants treated at 22 °C in the dark for 1 h after a 7 °C pretreatment (Fig. 4F). Under these conditions, the effect of cold priming on phototropism mostly disappeared (Fig. 4G), indicating ‘de-priming’. These results indicate that phot2-mediated cold priming is based on the temperature information just before dawn and that this information affects subsequent phototropism for the next several hours. Importantly, thermal memory can be rewritten until BL irradiation.

## Discussion

In this study, we determined that phot2 mediates hour-scale cold priming to promote stomatal opening and phototropism in Arabidopsis. Thus, phot2 appears to play a role as a thermosensor not only for chloroplast relocation, but also for stomatal opening and phototropism. Therefore, Arabidopsis uses both phot1 and phot2 as photoreceptors but only phot2 as a thermosensor for various physiological responses under fluctuating light and temperature conditions.

In the present study, we developed dawn-mimicking conditions to analyse stomatal opening and phototropism. In these conditions, BL irradiation and a temperature rise were applied simultaneously to the plants. However, actual dawn in nature involves more complex conditions, such as various combinations of BL irradiation timing and temperature rise timing. Under such complicated conditions, physiological responses are likely to be optimized. Indeed, as a preliminary experiment, we tested a 7 °C pretreatment under BL irradiation for 1 h before shifting the temperature to 22 °C (Supplementary Fig. S2). This 7 °C pretreatment under BL irradiation delayed the onset of phototropism while resulting in a higher bending velocity, similar to the cold-priming effect (Supplementary Fig. S2). Although the delayed onset may be explained by involvement in a refractory state of phototropism (Haga and Sakai, 2023), further studies will be needed to understand the underlying mechanism. Overall, the modulation of dawn-mimicking conditions could facilitate the study of elements within physiological responses.

Similar to the mechanism underlying how phot perceives temperature using its LOV2 domain in *M. polymorpha* (Fujii *et al.*, 2017), Arabidopsis phot2 appears to use the temperature sensitivity of the reversion of its LOV2 domain for thermosensing. Based on our measurements of thermal reversion, thermosensing via LOV2^phot2^ occurs on a timescale of less than 1 min. Similarly, phot1 also undergoes thermal reversion of its LOV2 domain in Arabidopsis (Christie, 2007), and the half-life of the active form of LOV2^phot1^ changes in a temperature-dependent manner (Okajima *et al.*, 2012) (Fig. 4B). Given the temperature sensitivity of the reversion of LOV2^phot1^, phot1 is also a potential thermosensor in Arabidopsis. Phot1-related signaling pathways for stomatal opening and phototropism were not activated at 7 °C (Fig. 3A–F), so we cannot conclude that phot1 functions as a thermosensor based on the current findings. However, we cannot rule out the possibility that phot1 functions as a thermosensor in other physiological responses, as our *in vitro* analysis suggested conditions under which phot1 can function as a thermosensor (Figs 2C, D, 4B). Additionally, in the phototropic response, *phot2-1* did not show obvious cold priming but exhibited a similar trend to the WT, with a higher degree of phototropism at 7 °C than at 22 °C (Fig. 1K, N). This may imply a thermosensor function for phot1. A previous study reported that the photosensitivity of LOV2^phot1^ is higher than that of LOV2^phot2^ at 20 °C under light intensities below 100 µmol m^–2^ s^–1^ of BL; for example, under 10 µmol m^–2^ s^–1^ of BL, 80% of LOV2^phot1^ was in the active form compared with 30% for LOV2^phot2^ (Okajima *et al.*, 2012). Based on these previous data, we speculate that phot1 functions as a thermosensor under dim BL. Indeed, we observed phot2-independent cold priming in phototropism under weaker BL conditions (1 µmol m^–2^ s^–1^) (Supplementary Fig. S3). Even if this phot2-independent cold priming is mediated by thermosensing through phot1, our present data suggest (Fig. 3E, F) that an unknown regulatory protein(s) other than NPH3 and RPT2 is involved in this phot2-independent cold priming. Further study is needed to ascertain the role of phot1 as a thermosensor in Arabidopsis.

Although phot2-based thermal memory operates on a timescale of less than 1 min, the phot2-mediated cold-priming effect was observed after some time had passed under light conditions: 30–50 min for stomatal opening and 4 h for phototropism (Fig. 1E, G, K). Phot2-mediated cold-priming could amplify the signal intensity to regulate stomatal opening and phototropism, rather than accelerate the onset of these responses. The initial small signal via the cold priming at the molecular level would be gradually amplified to promote stomatal opening and phototropism at the cellular and tissue levels, respectively. The difference between the cellular and tissue levels may explain the varying observation times for the cold-priming effect between stomatal opening and phototropism. Alternatively, we cannot exclude the possibility that phot2 might be post-translationally modified in an unknown manner under cold conditions in darkness, contributing to the increased and sustained effect. If such phot2 modification exists in Arabidopsis, based on previous reports on phot1 and phot2, ubiquitination and sumoylation may be potential post-translational mechanisms (Deng *et al.*, 2014; Łabuz *et al.*, 2021). Nevertheless, the detailed behavior and function of phot2 under cold conditions remain to be elucidated.

Phot-mediated physiological responses are induced under BL conditions. However, to date, the analysis of thermal reversion of the LOV domain has been technically limited in the dark (Okajima *et al.*, 2012). In the present study, we developed a fluorescence-based method to analyse thermal reversion under BL conditions (Fig. 2). This method facilitates understanding of LOV-mediated thermosensing functions of phot under BL conditions at various temperatures.

As lower temperatures inhibit all biochemical activities in the cell, phot2-mediated cold priming facilitates optimal stomatal opening and phototropism in response to light at dawn, allowing for efficient photosynthesis early in the day. Night-time temperatures fluctuate due to radiative cooling, which depends on the amount of cloud cover. Importantly, the extent of cloud cover also affects the intensity of sunlight at dawn: sunlight is less intense when conditions are cloudy at dawn and more intense when conditions are clear at dawn. In other words, although there may be exceptions, the relative change in temperature at night correlates with the intensity of sunlight at dawn. Given this correlation, phot2 may predict sunlight intensity (i.e. cloud cover) at dawn based on its memory of the temperature just before dawn and may adjust stomatal opening and the phototropic response, with weaker responses at dawn under cloudy conditions and stronger responses at dawn under clear conditions. Importantly, phot2-mediated thermal memory can be rewritten until the period just before dawn (Fig. 4F, G). This rewritable thermal memory would allow plants to modulate the strength of their BL responses at dawn using temperature information from just before dawn, even if there were transient temperature changes earlier in the night (e.g. midnight) that would be irrelevant for predicting conditions at daybreak.

Almost all organisms are exposed to lower temperatures at night than during the day; thus, daily cold priming likely plays a role in various organisms. Almost all photoreceptors are thought to undergo thermal reversion that could in theory be used for temperature sensing, suggesting that daily cold priming mediated by thermosensitive photoreceptors may occur during various light responses at dawn.

## Supplementary data

The following supplementary data are available at *JXB* online.

Fig. S1. Photosynthesis does not mediate the cold priming of stomatal opening.

Fig. S2. A delayed cold priming of phototropism induced by BL irradiation under low-temperature conditions.

Fig. S3. Cold priming of phototropism in the WT and *phot2-1* mutant under dim BL conditions.

Table S1. Primers used in this study.

eraf040_suppl_Supplementary_Figures_S1-S3

## Data Availability

The authors declare that all data supporting the finding of this study are available within the paper or are available from the corresponding author upon request.
