# Supplementary material for: Phototropin 2 mediates daily cold priming to promote light responses in Arabidopsis
Source: J Exp Bot. 2025 Jan 30;76(8):2309–19. doi: 10.1093/jxb/eraf040 (PMC12116169; doi:10.1093/jxb/eraf040)
Supplement: eraf040_suppl_Supplementary_Figures_S1-S3 [file eraf040_suppl_supplementary_figures_s1-s3.pdf]

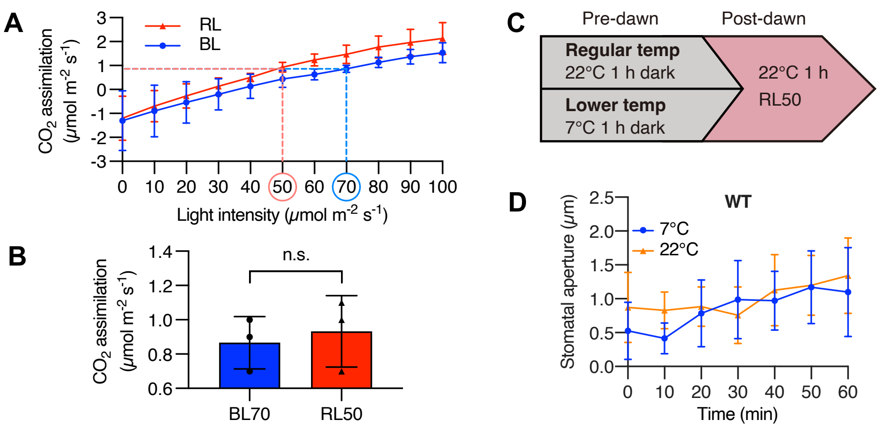

Supplementary Fig. S1. Photosynthesis does not mediate the cold priming of stomatal opening.

(A) CO_2 assimilation under various intensities of blue light (BL) and red light (RL) (n = 3). (B) No significant difference in CO_2 assimilation was detected between BL at 70 μmol m^{-2} s^{-1} (BL70) and RL at 50 μmol m^{-2} s^{-1} (RL50). (C) Diagram of the dawn-mimicking conditions used to induce stomatal opening with red light (50 μmol m^{-2} s^{-1}; RL50). (D) Stomatal opening without cold priming in the WT under the dawn-mimicking conditions illustrated in (C) (n = 7). Values are means ± SD (A,B,D). n.s. indicates no significant difference (P = 0.4226), as determined by Student's t-test (B,D).

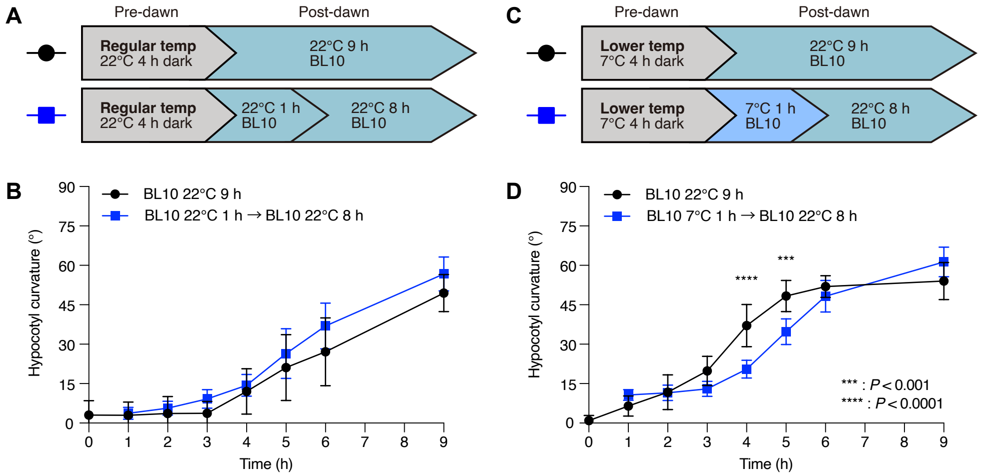

Supplementary Fig. S2. A delayed cold priming of phototropism induced by BL irradiation under low-temperature conditions.

(A,C) Diagram of dawn-mimicking conditions used to induce phototropism with 10 µmol m^{-2} s^{-1} of blue light (BL10). Temp, temperature. (B) Phototropism in the WT after dawn under the dawn-mimicking conditions illustrated in (A) (n = 5). (D) Phototropism and cold priming in the WT after dawn under the dawn-mimicking conditions illustrated in (C) (n = 5). Values are means ± SD (B, D). Asterisks indicate significant differences (Sidak's multiple comparisons test) (B,D).

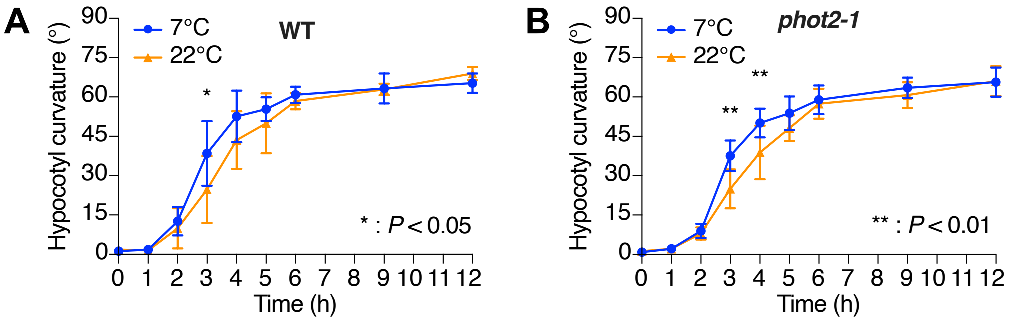

Supplementary Fig. S3. Cold priming of phototropism in the WT and *phot2-1* mutant under dim BL conditions.

Phototropism and cold priming in the WT (A) and *phot2-1* (B) after dawn under the dawn-mimicking conditions shown in Fig. 1I with weaker BL (1 µmol m^{-2} s^{-1}) (n = 5). Values are means ± SD. Asterisks indicate significant differences (Sidak's multiple comparisons test).
